# Inhibition of HIV-1 Infection in *Ex Vivo* Cervical Tissue Model of Human Vagina by Palmitic Acid; Implications for a Microbicide Development

**DOI:** 10.1371/journal.pone.0024803

**Published:** 2011-09-19

**Authors:** Xudong Lin, Elena E. Paskaleva, William Chang, Alexander Shekhtman, Mario Canki

**Affiliations:** 1 Center for Immunology and Microbial Disease, Albany Medical College, Albany, New York, United States of America; 2 Department of Chemistry, State University of New York at Albany, Albany, New York, United States of America; 3 Palm Biologicals, LLC. Albany, New York, United States of America; University of Cape Town, South Africa

## Abstract

**Background:**

Approximately 80% of all new HIV-1 infections are acquired through sexual contact. Currently, there is no clinically approved microbicide, indicating a clear and urgent therapeutic need. We recently reported that palmitic acid (PA) is a novel and specific inhibitor of HIV-1 fusion and entry. Mechanistically, PA inhibits HIV-1 infection by binding to a novel pocket on the CD4 receptor and blocks efficient gp120-to-CD4 attachment. Here, we wanted to assess the ability of PA to inhibit HIV-1 infection in cervical tissue *ex vivo* model of human vagina, and determine its effect on *Lactobacillus* (*L*) species of probiotic vaginal flora.

**Principal Findings:**

Our results show that treatment with 100–200 µM PA inhibited HIV-1 infection in cervical tissue by up to 50%, and this treatment was not toxic to the tissue or to *L. crispatus* and *jensenii* species of vaginal flora. *In vitro*, in a cell free system that is independent of *in vivo* cell associated CD4 receptor; we determined inhibition constant (Ki) to be ∼2.53 µM.

**Significance:**

These results demonstrate utility of PA as a model molecule for further preclinical development of a safe and potent HIV-1 entry microbicide inhibitor.

## Introduction

Worldwide, sexual transmission remains the principal route of HIV transmission accounting for approximately 80% of all new infections, and women bear a disproportionate burden of people living with HIV (UNAIDS, 2010) [1]. In the absence of an effective vaccine, there is an urgent need to supplement currently available strategies with novel therapeutics including microbicides that are aimed at preventing sexual transmission of HIV. Viral entry inhibitors are ideally suited for use in microbicide formulations [2], however currently there are no clinically approved micobicide or trials that specifically include CD4 inhibitors of virus entry into the host (for a full list see www.avac.org).

In our search for novel inhibitors of HIV-1, we investigated a large number of natural products, and from *Sargassum fusiforme* we isolated and identified palmitic acid (PA) as a natural small molecule that blocked virus entry [3], [4], [5]. We reported that palmitic acid is a specific CD4 fusion inhibitor of both X4 and R5 HIV-1 entry, which also efficiently inhibited virus-to-cell and cell-to-cell fusion, and it did not internalize CD4 receptor or perturb lipid rafts [6]. PA bound to the CD4 receptor specifically, with dissociation constant (K_d_) of ∼1.5 µM, and this binding was *via* PA's hydrophobic methyl and methelene groups located away from the carboxyl end, which functions by blocking efficient pgp120-to-CD4 attachment and fusion [6], [7]. We also showed that PA occupies a novel hydrophobic cavity on the CD4 receptor that is constrained by amino acids Phe52-to-Leu70 [7], which encompass residues that have been previously identified as a region critical for gp120 binding [8], [9].

In the present report we wanted to ascertain PA's utility for microbicide development by testing its ability to block HIV-1 infection in *ex vivo* model of vaginal mucosa, its effect on *Lactobacillus* species present in normal vaginal flora, and PA's *in vitro* inhibition constant independent of variable *in vivo* CD4 expression.

## Results

**Figure 1:**
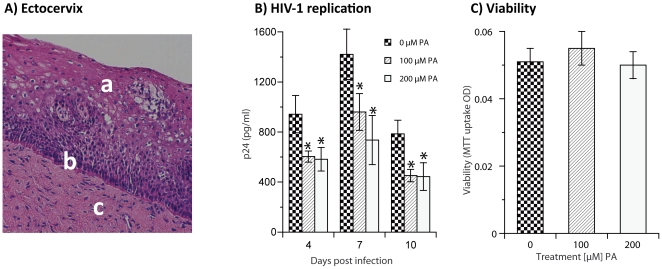
Inhibition of HIV-1 infection in human cervix model of vaginal mucosa. 3 mm^3^ biopsy punches of the ectocervix tissue samples from premenopausal women with conditions not involving the cervix were processed within 1–3 hours after surgery and directly cultured in 48 well plates in 300 µl/well DMEM/F12 media. (A) Paraffin embedded, and hematoxylin and eosin (H&E) stained sections of the uninfected ectocervix tissue were identified to be composed of (a) stratified squamous epithelial cell layer, (b) basal epithelial layer, and (c) submocosa, which was visualized on an Olympus BX41 Altra 20 Soft Image System, 100× magnification. (B) Replicates (n = 6) of tissue were treated for 24 h with 0, 100, or 200 µM PA, and then infected with 2×10^5^ p24/ml cell-free HIV-1 BaL in 300 µl for 16 h. Tissues was washed 3 times to remove the virus, and returned to culture with each respective treatment for the duration of the experiment. At the indicated time points, HIV-1 replication was tested by p24 ELISA, and repeated measures ANOVA was used to calculate statistical significance (*) between groups. (C) At day 10 after infection, tissue was collected and viability determined by the MTT assay. Representative of three experiments, all data are mean ± SD.

Based on our previous results demonstrating that PA is a specific HIV-1 entry inhibitor and a potential lead molecule for further development [6], [7], we wanted to examine the practical utility of PA to inhibit HIV-1 infection in a human cervical tissue *ex vivo* model of vaginal mucosa. This model closely resembles the vaginal epithelial layer that mimics *in vivo* conditions for HIV sexual transmission and infection [10], [11], [12], and it has been established for evaluating potential microbicide candidates [13], [14], [15]. Light microscopy examination of the uninfected and paraffin embedded sections from 3 mm^3^ biopsy punches of the cervix revealed that the tissue architecture was preserved (Fig. 1A). Although both X4 and R5 viruses are sexually transmitted, R5 viruses predominate early in infection and may be more easily transmissible than X4 viruses [12], [14]. Tissue was treated with 0, 100 or 200 µM PA, and then tested for inhibition of productive HIV-1 R5-tropic BaL infection by p24 ELISA (Fig. 1B). Measurements of HIV-1 p24 antigen levels in PA untreated cell-free tissue culture supernatants (0 µM) revealed a peak of p24 production on day 7 that measured 1421 pg p24/ml. This represented an increase from 943 pg p24/ml on day 4, which was followed by a gradual decline on day 10, to 785 pg p24/ml. Increasing p24 values indicated productive and ongoing HIV-1 infection, and *de novo* viral synthesis. In contrast, treatment with 100 µM PA, significantly reduced HIV-1 replication to 604, 960, and 452 pg p24/ml on days 4, 7, and 10, respectively (p = 0.04, repeated measures ANOVA). Compared to PA untreated tissue, this reduction in HIV-1 replication corresponded to a calculated 36, 32, and 42% inhibition of HIV-1 infection. Similar results were obtained for treatment with 200 µM PA, which also significantly reduced HIV-1 infection by 38, 48, and 43% on day 4, 7, and 10, respectively. However, there appeared to be no significant difference between 100 and 200 µM PA treatment. Tissue toxicity was measured on day 10 after infection, by MTT viability assay, which demonstrated an absence of toxicity (p = 0.64, one-way ANOVA) (Fig. 1C). Tissue infection with X4 HIV-1 was less productive, however inhibition by PA was similar to that of R5 infection (not shown).

**Figure 2:**
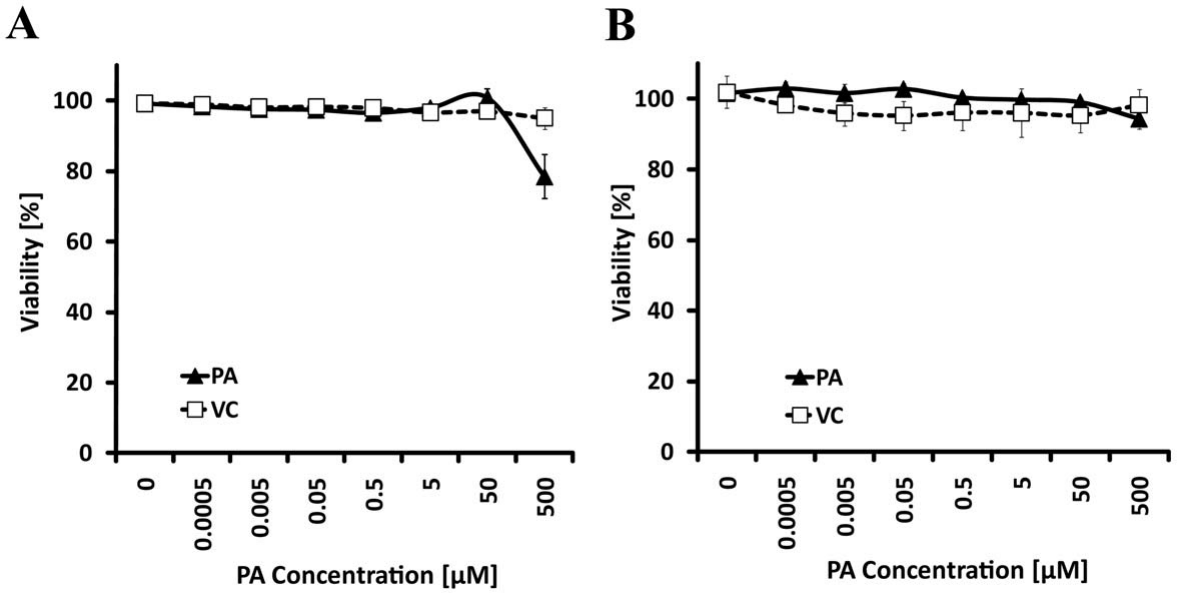
*Lactobacillus* toxicity assay. *L. crispatus* (A) and *jensenii* (B) species were tested for toxicity against increasing concentrations of PA that was diluted in vehicle control (VA), 0.5% DMSO highest concentration. Dilutions of PA or VC were plated in triplicate on 96-well plates in 100 µl volume, and bacterial species grown in MRS medium were diluted and added to a final volume of 200 µl. After 24 hours incubation, bacterial cultures were read in a spectrophotometer at OD_490_ and % viability was calculated from untreated cultures (0 µg PA) that was taken as 100% viability. Bars indicate ± SD of three separate experiments.

*In vivo*, normal vaginal flora consists predominantly of common probiotic *Lactobacillus* bacterial species [16] that a successful topical microbicide should not be toxic to, as was outlined in screening algorithm for testing of preclinical topical microbicides [17]. To determine potential compound toxicity, we tested PA treatment on common *L. crispatus* and *jensenii* species normally present in vaginal tract (Fig. 2 A and B). Increasing concentrations of PA were incubated for 24 hours in presence of either *L. crispatus* (Fig. 2A) or *jensenii* (Fig. 2B), and % viability was calculated from 0 µM PA (no treatment) that was taken as 100% viability. *L. crspatus* showed viability over 96% with PA concentrations of up to 50 µM, which decreased to 78.5% with 500 µM PA treatment. *L. jensenii* remained viable over 94% with up to the highest treatment of 500 µM PA. Vehicle control (VC) was also not toxic to either species that remained viable over 95%. Based on these results we conclude that PA may be considered suitable for further topical microbicide evaluation and development.

**Figure 3:**
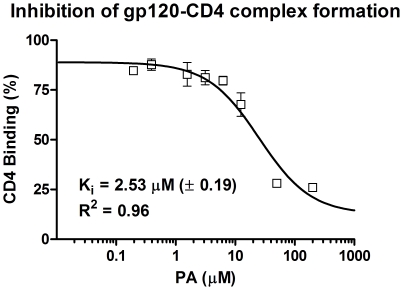
PA inhibition constant for gp120-to-CD4 complex formation. Inhibition of gp120-CD4 complex formation was investigated by gp120 capture ELISA. Envelope gp120 (IIIB) protein was captured on 96 well flat bottom plates, washed, and incubated in the presence of CD4-biotin alone or in the presence of increasing concentrations of PA, as indicated. Strepavidin-HRP was added, and then developed by addition of o-Phenylenediamine dihydrochloride (OPD) substrate. Colorimetric reaction was stopped by adding 1 N HCl, and read at 490 nm. % CD4 binding was calculated from gp120-CD4 complex formation in the absence of PA inhibitor, and inhibition constant, K_i_, was calculated utilizing previously published equation [18]. Representative of three experiments, all data are mean ± SD.

Peripheral blood lymhocytes (PBL) and macrophages (Mφ's) are primary targets for *in vivo* HIV-1 infection and replication in systemic circulation as well as in vaginal submucosa, and previously we demonstrated ability of PA treatment to inhibit ongoing virus replication in these physiologically relevant cells [6]. However, PA inhibited HIV replication in PBL with approximately 10-fold greater efficacy as compared to inhibition in Mφ's [6]. Because CD4 cell surface HIV receptor expression varies between different cell types, here we wanted to ascertain PA's inhibition efficacy that is independent of *in vivo* cell surface expression. We utilized *in vitro* gp120-to-CD4 capture ELISA to determine PA's inhibition constant (Ki) (Figure 3). Envelope gp120 (IIIB) protein was captured on 96 well plates, washed, and incubated in the presence of CD4-biotin alone or in the presence of serial dilutions of PA as indicated. Percent CD4 binding was calculated from gp120-CD4 complex formation in the absence of any inhibitor. Inhibition constant, K_i_, was calculated by using the equation K_i_ = IC_50_/(1+[CD4]/K_d_) [18], based on IC_50_ concentration of bound CD4, [CD4] = 50 nM, and CD4 binding affinity for gp120, K_d_ = 5 nM, which is consistent with and validated our *in vitro* K_d_ value of 1.5 µM for CD4 binding [6].

## Discussion

In this *ex vivo* model of female genital mucosa that simulates damaged epithelium and allows for maximal virus infection and replication [19], our data clearly demonstrate that the 100–200 µM PA treatment inhibits productive R5 HIV-1 infection by up to 48%, and that the PA treatment is not toxic to tissue or to probiotic *Lactobacillus* species tested here. Considering that PA was not formulated into a topical delivery vehicle may have imposed suboptimal tissue penetration and HIV inhibition in the underlying submucosa containing PBL and Mφ cells. Previously we demonstrated ability of PA treatment to inhibit ongoing virus replication in these physiologically relevant cells, however, inhibition was more efficient in PBL as compared to Mφ's [6]. The reason for differences in inhibition between tissue, PBL and Mφ's is not clear, however it may indicate variable CD4 receptor cell surface expression, and intrinsically different number of primary cells in each tissue biopsy. This proposition is logical considering that PA has to bind directly to the CD4 receptor to inhibit HIV-1 entry, and therefore quantitatively different CD4 expression will affect PA's inhibition efficacy. To avoid this degree of difference of *in vivo* primary cell number and CD4 expression, we tested *in vitro* PA's inhibition efficacy (K_i_) to block gp120-to-CD4 attachment (Figure 3). The measured K_i_ was ∼2.53 µM, which is in close agreement with previously published CD4 dissociation constant of ∼1.5 µM [6].

Although there are several candidate microbicides in clinical trials [20], PA is a novel class of small molecules with bi-functional mode of action that specifically targets and binds to the CD4 receptor *via* its hydrocarbon chain, and blocks efficient gp120-to-CD4 fusion *via* its carboxyl end [6], [7]. Targeting the human CD4 receptor as opposed to viral envelope or other viral proteins, offers the advantage of removing selective pressure on the virus to quickly mutate and bypass the imposed restriction.

Current results from The Center for the AIDS Program of Research in South Africa (CAPRISA) clinical trial have demonstrated that micobicide application of reverse transcriptase (RT) tenofovir gel, prevented HIV infection by up to 39% overall, and by 54% in women with high gel adherence [21]. Considering that nucleotide RT inhibitor prevents provirus transcription and not virus entry, we postulate that combination with an entry inhibitor such as PA may eliminate HIV infection with more robust efficacy. The idea of combing therapeutics that target different stages of virus life cycle is logical as it has been effectively demonstrated with HAART therapy in AIDS patients.

PA's toxicology screening, breadth of primary HIV inhibition, and gel formulations are currently under investigation.

## Materials and Methods

Palmitic acid (Sigma) was solublized at 100 mM in ethanol (EtOH) [22] and stored at −20°C. Working aliquots were kept at 4°C for up to 4 weeks.

Written informed consent approved protocol by Albany Medical College (AMC) Institutional Review Board (IRB) was obtained from all participants involved in the study, which was performed at the AMC, Albany New York. 3 mm^3^ biopsy punches of the ectocervix tissue samples were obtained from premenopausal women with conditions not involving the cervix, and were processed within 1–3 hours after surgery. Tissue was cultured in a nonpolarized manner as previously described [10], [19], in 48 well plates in 300 µl/well DMEM/F12 media (Invitrogen) supplemented with 10% FBS for the duration of the experiment. Tissue was treated with increasing concentrations of PA or 10^−6^ M ddC, and infected with HIV-1 R5 BaL at 0.3 multiplicity of infection (MOI).

*Lactobacillus crispatus* and *jensenii* species were purchased from American Type Culture Collection (ATCC), number 33820 and 25258, respectively. Bacterial toxicity assay was performed under facultative anaerobic conditions as previously described [17]. Briefly, bacterial species were grown in MRS medium and plated on 96 well plates containing increasing concentrations of PA. Penicillin-streptomycin solution (Sigma) at 1.25 U/ml and 1.25 µg/ml, respectively, was used as positive toxicity control. After 24 hours, bacterial culture growth was determined by measurement at OD_490_ using a 96 well SpectraMax L plate reader (Molecular Devices).

HIV-1 X4-tropic molecular clone NL4-3, which expresses all known HIV-1 proteins [23]; the R5-tropic molecular clone 81A [24], which has R5 BaL Env sequences on the backbone of NL4-3; and R5 BaL molecular clone were all obtained from the HIV AIDS Research and Reference Reagent Program. Macrophage HIV-1 R5-tropic isolate BaL was prepared as previously described [5], and cell-free viral stock was quantified for HIV-1 p24 core antigen content by enzyme-linked immunosorbent assay (ELISA, AIDS Vaccine Program, NCI-Frederick). Potential tissue or cell toxicity due to PA treatment was measured after infection by the MTT assay (Promega), as previously described [10].

Percent CD4 binding was investigated by CD4-to-gp120 capture ELISA, in accordance with manufacturer's instructions (ImmunoDiagnostics, Inc., MA), and as previously described [6]. Briefly, envelope gp120 (IIIB) protein (ImmunoDiagnostics, Inc., MA) was captured on 96-well plates, washed, and incubated in the presence of 50 nM biotin-conjugated sCD4 (Immunodiagnostics) alone or in the presence of serial dilutions of PA, as indicated. Strepavidin-HRP was added, and then developed by addition of OPD substrate. Colorimetric reaction was stopped by adding 1 N HCl, and read at 490 nm. Percent CD4 binding was calculated utilizing previously published formula [18].
